# Breeding values, stability, and heterotic grouping of Asian *Saccharum spontaneum* collection for parental selection in sugarcane program

**DOI:** 10.3389/fpls.2026.1923174

**Published:** 2026-09-28

**Authors:** Chao-Hua Xu, Farrag F. B. Abu-Ellail, LiPing Zhao, Jianwen Li, Xingdong Zhao, Yadong Bai, Yunfei Ma, Lihua Yang, Hai-Rong Huang, Sheng Xian Chen, Xin Lu

**Affiliations:** 1 State Key Laboratory of Tropical Crop Breeding, Sugarcane Research Institute, Yunnan Academy of Agricultural Sciences, Yunnan Key Laboratory of Sugarcane Genetic Improvement, Kaiyuan, China; 2 Breeding and Genetics Department, Sugar Crops Research Institute, Agriculture Research Center, Giza, Egypt; 3 Agricultural and Technology Services Center of Yuanjiang County of Yuxi Prefectural, Yuanjiang, China; 4 Sugarcane Research Institute, Guangxi Academy of Agricultural Sciences, Nanning, Guangxi, China

**Keywords:** AMMI, heterotic grouping, MGIDI, ratoon performance, *Saccharum spontaneum*, stability, WAASB

## Abstract

Sugarcane breeding is constrained by a narrow genetic base, limiting progress in ratooning ability, stress tolerance, and climate resilience. Although *Saccharum spontaneum* harbors valuable genetic diversity, its systematic utilization in breeding remains limited by a lack of multi-environment evaluations. Therefore, 100 Asian *S. spontaneum* clones were assessed across six environments (two locations × three crop cycles) during 2022–2025 using an integrated framework that combined BLUP breeding values, AMMI-based stability indices (ASV, GSI, HMRPGV, WAASB), and the multi-trait selection index MGIDI. Heritability, repeatability, and genetic correlations were estimated to support selection decisions. Moderate heritability was observed for stalk diameter and stalk height, while juice Brix and stalk weight showed low heritability. The clones were classified into four heterotic groups; only three clones (C059, C086, and C089) met both high-performance and high-stability thresholds, forming Group A (elite + stable). Group B (high performance, low stability) included 22 clones, Group C (low performance, high stability) included 22 clones, and Group D (moderate) comprised the remaining 53 clones. Repeatability from first to second ratoon was remarkably high (≥0.90) for most morphological traits, except stalk weight (0.07). Genetic correlations revealed a trade-off between juice Brix and internode length, and a positive association between tillering rate and fresh biomass. Crossing strategies should prioritize Group A × Group B and crosses between geographically distant origins (e.g., Yunnan × Hainan) to maximize heterosis. The three elite clones are recommended as immediate parents for climate-adaptive breeding programs.

## 1 Introduction

Sugarcane (*Saccharum* spp.) supplies over 80% of the world’s sugar and is an increasingly important bioenergy feedstock. However, modern cultivars suffer from an extremely narrow genetic base, limiting progress for complex traits such as abiotic stress tolerance and climate resilience (Jackson, 2005; Zhang and Yang, 2024; Abu-Ellail et al., 2026). To overcome this constraint, breeders have turned to wild relatives, among which *Saccharum spontaneum* L. stands out as the most variable wild species in the genus; it is widely recognized for high tillering, strong ratooning ability, and tolerance to drought, waterlogging, and salinity (Da Silva et al., 2018; Abu-Ellail et al., 2025). Moreover, it has been successfully used as a donor of genes for vigor, disease resistance, and stress tolerance in commercial varieties (Xu et al., 2020; Vinu et al., 2025), and consequently, it is increasingly viewed as essential for developing high-biomass ‘energy canes’ and climate-resilient cultivars.

China and neighboring Southeast Asian countries are major diversity centers for this species. Xu et al. (2020) demonstrated that Southwest China, particularly Yunnan Province, harbors the highest genetic diversity of *S. spontaneum*, and that geographical origin strongly correlates with genetic structure, thereby providing a rational basis for forming heterotic groups based on provenance and performance. Despite this potential, the systematic use of *S. spontaneum* in sugarcane breeding remains limited; multi-environment phenotypic evaluations of large collections are scarce, partly because the species is classified as a noxious weed in several countries, which restricts field experimentation (Da Silva et al., 2018). In fact, most published studies focus on molecular diversity (Fan et al., 2013) or single-trait (mainly disease) resistance, whereas comprehensive multi-trait, multi-environment assessments of breeding value are lacking. Furthermore, quantitative information on clone performance under contrasting climatic conditions, information essential for climate-adaptive breeding, remains scarce (Scordia et al., 2015; Vinu et al., 2025).

Genotype-by-environment (G×E) interaction is a major obstacle in multi-environment trials because it can conceal true genetic potential (Appunu et al., 2024). Several statistical tools help dissect such interactions: the Additive Main effects and Multiplicative Interaction (AMMI) model captures G×E patterns, and when combined with stability indices such as AMMI Stability Value (ASV; Liao et al., 2026), Genotype Selection Index (GSI; Durai et al., 2025), and Harmonic Mean of Relative Performance of Genotypic Values (HMRPGV; Resende, 2016), it allows quantitative ranking of genotypes by both mean performance and stability. More recently, the Weighted Average of Absolute Scores (WAASB) index (Olivoto et al., 2019) has been introduced as a unified metric that combines the strengths of AMMI and Best Linear Unbiased Prediction (BLUP), providing an integrated measure of stability. Selecting parents with high breeding values (genotypic values) for multiple target traits is equally crucial; BLUP is a standard method for estimating BV, as it accounts for the multi-environment data structure and provides robust estimates of additive genetic potential (Piepho et al., 2008). Moreover, when multiple correlated traits must be improved simultaneously, integrated multi-trait selection approaches are required. The Multi-trait Genotype-Ideotype Distance Index (MGIDI) index (Olivoto and Nardino, 2021) overcomes limitations of classical linear indices by using factor analysis to reduce dimensionality and selecting genotypes closest to a predefined ideotype, a feature particularly suitable when high Brix, high tillering, and adequate biomass are all desired in the same parent.

The main goal of this pre-breeding screening was to identify elite parental clones for use in a sugarcane breeding program, since heterotic grouping provides a means to exploit additive and non-additive genetic effects through reciprocal recurrent selection (Mendes de Paula et al., 2020).To this end, 100 Asian *S. spontaneum* clones were evaluated across six environments. The specific steps involved: (i) estimating genetic parameters (variance components, heritability, expected genetic advance, and BLUP breeding values) and repeatability across crop cycles; (ii) dissecting G×E via AMMI-based stability indices (ASV, GSI, HMRPGV, WAASB); (iii) applying MGIDI for multi-trait selection; (iv) classifying clones into heterotic groups; and (v) proposing crossing strategies. Ultimately, this research bridges germplasm characterization with practical breeding decisions, ensuring the effective use of *S. spontaneum* genetic resources to enhance juice quality (Brix), biomass production, tillering rate, and ratooning ability in future sugarcane hybrids. The evaluation across diverse locations and crop cycles ensures that the selected parents possess broad adaptation.

## 2 Materials and methods

### 2.1 Plant material and experimental design

One hundred elite *S. spontaneum* clones were obtained from the China National Nursery of Sugarcane Germplasm Resources (CNNSGR), Yunnan Sugarcane Research Institute, Kaiyuan, China. The clones originated from 12 geographical regions: Yunnan (36), Hainan (33), Guangdong (6), Fujian (6), Guizhou (5), Sichuan (5), Thailand (4), Vietnam (2), Hunan (1), Guangxi (1), Laos (1), and India (1) (**Table 1**). The field experiment was conducted over three crop cycles namely, plant cane, first ratoon, and second ratoon at two Yunnan locations (Kaiyuan and Yuanjiang), with cycle durations of 10, 9, and 9 months, respectively. Each unique combination of location and crop cycle was treated as a distinct environment, resulting in six environments (two locations × three crop cycles). Planting and harvesting dates are given in **Table 2**.

**Table 1:** List of the 100 *Saccharum spontaneum* clones used in the study.

Code	Clone name	Code	Clone name	Code	Clone name	Code	Clone name
C1	Fujian 79-1-9	C26	Hainan 92-12	C51	Hunan 2012-151	C76	Yunnan 8
C2	Fujian 79-2-9	C27	Hainan 92-16	C52	India 2	C77	Yunnan 82-114
C3	Fujian 87-1-8	C28	Hainan 92-25	C53	Laos 2	C78	Yunnan 82-120
C4	Fujian Jinshan	C29	Hainan 92-31	C54	Sichuan 79-1-17	C79	Yunnan 82-127
C5	Fujian Putian	C30	Hainan 92-32	C55	Sichuan 79-1-8	C80	Yunnan 82-129
C6	Fujian Xianyou 2	C31	Hainan 92-33	C56	Sichuan 79-2-9	C81	Yunnan 82-14
C7	Guangdong 10	C32	Hainan 92-34	C57	Sichuan 89-3	C82	Yunnan 82-140
C8	Guangdong 23	C33	Hainan 92-50	C58	Sichuan Neijiang 27	C83	Yunnan 82-16
C9	Guangdong 28	C34	Hainan 92-52	C59	Thailand 1	C84	Yunnan 82-6
C10	Guangdong 29	C35	Hainan 92-54	C60	Thailand 2003-1	C85	Yunnan 82-63
C11	Guangdong 53	C36	Hainan 92-56	C61	Thailand 2003-2	C86	Yunnan 82-65
C12	Guangdong 81	C37	Hainan 92-58	C62	Thailand 98T-266	C87	Yunnan 82-73
C13	Guizhou 78-1-30	C38	Hainan 92-59	C63	Vietnam 2	C88	Yunnan 82-77
C14	Guizhou 78-2-13	C39	Hainan 92-61	C64	Yuenan 1	C89	Yunnan 82-78
C15	Guizhou 78-2-14	C40	Hainan 92-63	C65	Yunnan 1	C90	Yunnan 82-79
C16	Guizhou 78-2-26	C41	Hainan 92-66	C66	Yunnan 10	C91	Yunnan 82-86
C17	Guizhou 78-2-28	C42	Hainan 92-76	C67	Yunnan 2	C92	Yunnan 84-249
C18	Hainan 1	C43	Hainan 92-8	C68	Yunnan 2009-18	C93	Yunnan 84-266
C19	Hainan 2011-23	C44	Hainan Haikou	C69	Yunnan 2009-34	C94	Yunnan 84-268
C20	Hainan 2011-42	C45	Hainan Ledong 2	C70	Yunnan 75-1-4	C95	Yunnan 87-27
C21	Hainan 2011-44	C46	Hainan Lingshui 5	C71	Yunnan 75-1-8	C96	Yunnan 87-30
C22	Hainan 2011-56	C47	Hainan Lingshui 6	C72	Yunnan 75-2-23	C97	Yunnan 87-32
C23	Hainan 3	C48	Hainan Tongshi 7	C73	Yunnan 75-2-26	C98	Yunnan 89-281
C24	Hainan 92-10	C49	Hainan Yacheng 12	C74	Yunnan 75-2-36	C99	Yunnan 89-282
C25	Hainan 92-11	C50	Hainan Yanglan 9	C75	Yunnan 76-3-18	C100	Yunrui 1-28

**Table 2:** Planting and harvesting dates for the plant cane, first ratoon, and second ratoon crops at Kaiyuan and Yuanjiang, representing the six environments (two locations × three crop cycles).

Crop cycle	Activity	Kaiyuan	Yuanjiang
Plant Cane	Planting	19 October 2022	9 September 2022
	Harvest	20 August 2023	13 July 2023
First Ratoon	Ratoon cut	20 August 2023	13 July 2023
	Harvest	23 May 2024	11 April 2024
Second Ratoon	Ratoon cut	23 May 2024	11 April 2024
	Harvest	19 February 2025	9 January 2025

The 100 clones were arranged in a randomized complete block design (RCBD) with three replications. Single-budded sets were planted in circular concrete rings (approximately 60 cm diameter × 40 cm height) installed in the field under open-air conditions without protective covering. Each ring was partially buried, with approximately 30 cm below the soil surface and 10 cm above, while the bottom remained completely open to allow unrestricted root penetration into the underlying natural soil; the concrete walls prevented lateral root interference between adjacent rings. The ring size (113 L) was chosen to minimize root restriction and approximate the root-zone volume available to field-grown plants (Poorter et al., 2012). A total of 600 rings were used (100 clones × 2 locations × 3 replicates), and the same rings were evaluated across three crop cycles (plant cane, first ratoon, and second ratoon). Each ring was filled with a 5-cm drainage layer of fine gravel at the bottom, followed by a potting mix consisting of sterilized field soil, sand, peat moss, and vermiculite in a 2:1:1:1 ratio (v/v). The potting mix was sterilized by autoclaving at 121°C for 20 minutes to eliminate soil-borne pathogens. The potting soil had a pH of 7.8, an organic matter content of 21.2 g kg^−1^, and total N, P, and K of 0.11, 0.15, and 1.2 g kg^−1^, respectively. Standard cultural practices for sugarcane, including irrigation and fertilizer application, were followed throughout the experiment. After emergence, four uniform seedlings were maintained per ring. This concrete ring system, installed directly in the field with an open bottom allowing root access to the subsoil, combines the advantages of lateral root isolation with natural soil conditions, making it suitable for initial screening of large germplasm collections prior to multi-location field trials.

### 2.2 Characterized traits

Eight agronomic and physiological traits were recorded across the three crop cycles (plant cane, first ratoon, and second ratoon), with details in **Table 3**. Juice Brix (Brix) and tillering rate (TR) served as primary selection criteria, followed by biomass-related traits (stalk height, diameter, weight, and fresh biomass).

**Table 3:** List of measured traits, abbreviations, units, and measurement protocols.

Trait	Abbreviation	Unit	Measurement protocol
Seeding rate *	SR	No. ring^−1^	Number of emerged seedlings per concrete ring (plant cane only).
Tillering rate	TR	%	Calculated at 120 days after planting (DAP) as (Total tillers – Main shoots)/Main shoots × 100.
Stalk height	SH	cm	Height of the tallest stalk from soil surface to apical meristem at harvest.
Stalk diameter	SD	mm	Measured at the mid-point of the third fully mature internode using a digital caliper.
Internode length	IL	cm	Average length of three mature internodes from the middle portion of the tallest stalk.
Stalk weight	SW	kg stalk^−1^	Fresh weight of a representative stalk after topping and leaf removal.
Fresh biomass	FB	kg ring^−1^	Total fresh weight of all above-ground biomass per concrete ring.
Juice Brix	Brix	%	Soluble solids percentage measured from crushed stalk juice using an electronic refractometer.

*Seeding rate (percentage of emerged buds) was recorded only in the plant cane cycle, 60 days after planting.

### 2.3 Climatic data and phenological stage definition

Monthly climatic data, mean temperature (°C), precipitation (mm), relative humidity (%), solar radiation (J·m^−2^·d^−1^), and wind speed (m·s^−1^), were collected from local meteorological stations (2022–2025) for two locations: Kaiyuan (23°42′ N, 103°15′ E) and Yuanjiang (23°36′ N, 101°59′ E). Data are presented in **Figure 1**. Based on crop durations of 10 months for plant cane and 9 months for each ratoon cycle (first and second ratoon), four phenological stages (establishment, tillering, grand growth, and early ripening) were defined according to sugarcane physiology (**Table 4**). For each stage, the following climate variables were calculated: total precipitation, mean temperature, growing degree days (GDD, base 10 °C), relative humidity, total solar radiation, and mean wind speed, to serve as environmental covariates for characterizing each environment.

**Figure 1:**
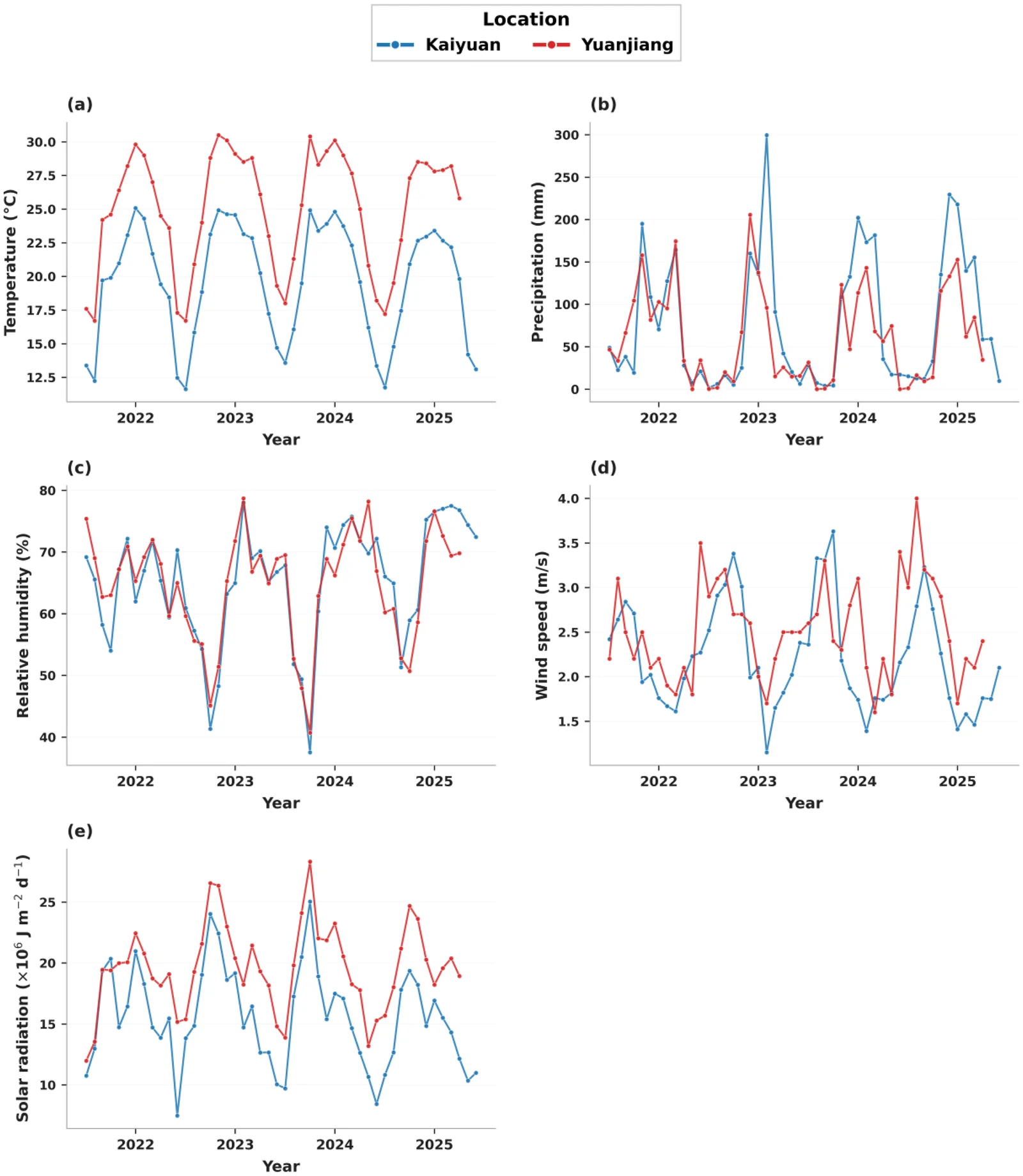
Monthly climate at Kaiyuan (blue) and Yuanjiang (red) from 2022 to 2025. The panels represent: **(a)** mean temperature (°C); **(b)** total precipitation (mm); **(c)** mean relative humidity (%); **(d)** mean wind speed (ms^−1^); and **(e)** solar radiation (Jm^−2^d^−1^). Precipitation is shown as grey bars, whereas the other variables are presented as lines. The x-axis is labeled “Year” with tick marks positioned at mid-year. Data were collected monthly and represent the average for each month.

**Table 4:** Phenological stages, duration [days after planting (DAP) and ratoon cut (DAR)], and associated primary traits for the plant cane (10 months) and ratoon cycles (first and second ratoon, 9 months each).

Stage	Description	Duration (DAP/DAR)	Associated trait(s)
I	Establishment	0 – 60	Seedling rate (plant cane only)
II	Tillering	61 – 120	Tillering rate (TR)
III	Grand growth	Plant cane: 121–240 Ratoon (first & second): 121 – 200	SH, SD, IL, SW, FB
IV	Early ripening	Plant cane: 241 – 300 Ratoon (first & second): 201 – 270	Juice Brix

### 2.4 Estimation of breeding values (EBVs)

#### 2.4.1 BLUP estimation, genetic parameters, and correlations

Best Linear Unbiased Prediction (BLUP) was used to estimate breeding values (genotypic values) of the 100 clones. A linear mixed model was fitted using Restricted Maximum Likelihood (REML) with the lmer function in the lme4 package (R version 4.6.0; R Core Team, 2026):


Y=μ+G+E+G×E+R(E)+ϵ

where Y is the phenotypic value, μ is the grand mean, G is the random clone effect, E is the fixed environment effect (location × crop cycle), G×E is the random interaction, R(E) is the random replicate within environment, and ϵ is the residual error. For traits measured only in plant cane (e.g., seeding rate), the model was simplified by removing the environment term. Clone random effects (G) were extracted as BLUP values; genotypic values were calculated as $\mu +\hat{g}$.

Variance components (σ^2^G, σ^2^G×E, and σ^2^ϵ) were extracted from the REML output. Broad-sense heritability was estimated as H^2^ = σ^2^G/(σ^2^G + σ^2^G×E + σ^2^ϵ). Genetic and phenotypic coefficients of variation were calculated as:


$$\text{GCV}\%=\left(\surd \sigma^{2}\text{G}/\text{overall mean}\right)\times 100.$$

$$\text{PCV}\%=\left(\surd \left(\sigma^{2}\text{G}+\sigma^{2}\text{G}\times \text{E}+\sigma^{2}\epsilon \right)/\text{overall mean}\right)\times 100.$$

Expected genetic advance (GA) under 5% selection intensity (k = 2.06) was $\text{GA}=\text{k}\times \surd \left(\sigma^{2}\text{G}+\sigma^{2}\text{G}\times \text{E}+\sigma^{2}\epsilon \right)\times {\text{H}}^{2}$, and genetic advance as a percentage of the mean was GAM% = (GA/overall mean) × 100.

Genetic correlations (rg) were estimated as the Pearson correlation of clone BLUP values for each trait pair; phenotypic correlations (rp) were calculated from raw plot means. All analyses were performed in R version 4.6.0 (R Core Team, 2026) using base functions, the tidyverse package for data manipulation, and openxlsx for output.

#### 2.4.2 Ratooning ability, relative performance, and repeatability

Ratooning ability (RA) was calculated for each clone and trait as $\text{RA}=\left({\bar{Y}}_{\text{r}}/{\bar{Y}}_{\text{p}}\right)\times 100$, where ${\bar{Y}}_{\text{r}}$ is the mean across the first and second ratoon crops and ${\bar{Y}}_{\text{p}}$ is the plant cane mean (Milligan et al., 1996). Relative performance (RP) was computed as: $\text{RP}=\left({\bar{Y}}_{\text{p}}+{\bar{Y}}_{{\text{r}}^{1}}+{\bar{Y}}_{{\text{r}}^{2}}\right)/3$. Higher RP values indicate superior average performance. RP values are presented using lollipop charts, following the approach of Masri and Amein (2015). Repeatability (R^2^) was estimated as the squared Pearson correlation between clone BLUP values for each trait across cycle pairs (plant cane vs. first ratoon, plant cane vs. second ratoon, and first vs. second ratoon) following Abu-Ellail et al. (2019). High R^2^ indicates consistent genotypic rankings; low R^2^ suggests strong genotype-by-cycle interaction requiring multi-cycle evaluation. The difference in repeatability (FR-SR minus PC-FR) was visualized with a lollipop chart; positive values denote higher repeatability from first to second ratoon.

### 2.5 Analysis of genotype × environment interaction

A preliminary screening was conducted across six environments (two locations × three crop cycles) to evaluate a large number of clones (n = 100) under representative yet limited conditions. Although the number of environments is modest, the six environments capture key sources of variation (location and ratoon cycle), thereby providing a reliable initial ranking for stability and performance.

#### 2.5.1 AMMI model

The additive main effects and multiplicative interaction (AMMI) model was applied to decompose the genotype-by-environment interaction (GEI). For each trait, the AMMI model is expressed as:


$${\text{Y}}_{\text{ij}}=\mu +{\text{G}}_{\text{i}}+{\text{E}}_{\text{j}}+\sum_{\text{k}=1^{\text{p}}}\left(\lambda_{\text{k}}\gamma_{\text{ik}}\delta_{\text{jk}}\right)+\epsilon_{\text{ij}}$$

where Y_ij_ is the mean performance of genotype i in environment j; μ is the grand mean; G_i_ is the main effect of genotype i; E_j_ is the main effect of environment j; λ_k_ is the singular value for the k-th interaction principal component axis (IPCA); γ_ik_ and δ_jk_ are the genotype and environment eigenvector scores for IPCA_k_; p is the number of significant IPCAs; and ϵ_ij_ is the residual error.

##### 2.5.1.1 AMMI Stability Value (ASV) was calculated using the first two IPCAs:


$${\text{ASV}}_{\text{i}}=\surd \left[{\left({\text{SS}}_{\text{i}}/{\text{SS}}_{2}\times \text{PC}1_{\text{i}}\right)}^{2}+{\left(\text{PC}2_{\text{i}}\right)}^{2}\right]$$

Where SS_1_ and SS_2_ are the sums of squares of the first and second IPCAs, and PC1_i_ and PC2_i_ are the scores of genotype i on the first and second IPCAs. Lower ASV indicates higher stability.

##### 2.5.1.2 Sum of absolute IPC Scores (SIPC) was calculated using SIPC_i_ = |PC1_i_| + |PC2_i_|

##### 2.5.1.3 Genotype Selection Index (GSI)

GSI combines the ranks of mean performance and ASV: GSI_i_ = Rank (Mean_i_) + Rank (ASV_i_)

Where Rank (Mean_i_) is the rank of genotype i in descending order of mean (highest mean = rank 1), and Rank (ASV_i_) is the rank in ascending order of ASV (lowest ASV = rank 1). Lower GSI indicates better combined productivity and stability.

##### 2.5.1.4 Harmonic Mean of Relative Performance of Genotypic Values (HMRPGV)


$${\text{HMRPGV}}_{\text{i}}=\left(1/\text{e}\right)\times \sum_{\text{j}=1^{\text{e}}}\left[1/\left({\text{BLUP}}_{\text{ij}}/\mu_{\text{j}}\right)\right]$$

Where e is the number of environments, BLUP_ij_ is the genotype mean in environment j, and μ_j_ is the environment mean. Higher values indicate better relative performance across environments. Analyses were performed in R 4.6.0 (R Core Team, 2026) using readxl and tidyverse for data handling, metan for AMMI, and ggplot2 for visualization.

#### 2.5.2 Parametric stability indices

##### 2.5.2.1 WAASB index

The Weighted Average of Absolute Scores (WAASB) was computed using the waas() function in the metan package R version 4.6.0; (R Core Team, 2026) to quantify stability independently of mean performance. The WAASB was calculated as:


WAASBi=(∑_{k=1}^{p}|IPCAik×EPk|)/(∑_{k=1}^{p}EPk)

Where IPCAik is the score of genotype i on the k-th interaction principal component axis, and EPk is the proportion of the total variation explained by that axis. WAASB measures the weighted distance of a genotype from the ideal stability point (the biplot origin); lower values indicate higher stability. Stability was assessed separately from mean performance, as the latter was evaluated using the MGIDI multi-trait index.

##### 2.5.2.2 Multi-Trait Genotype-Ideotype Distance Index (MGIDI)

MGIDI (Olivoto and Nardino, 2021) was used to select parents with high multi-trait performance. The ideotype was defined as the theoretical genotype with the maximum value for each trait, as higher values were desirable for all seven traits (stalk height, stalk diameter, internode length, stalk weight, fresh biomass, juice Brix, and tillering rate). All traits were assigned equal importance in the analysis. MGIDI was calculated using BLUP-adjusted clone means (averaged across environments), which account for environmental effects and provide more reliable genotypic estimates. Following factor analysis on clone means (two factors retained), MGIDI was computed as the Euclidean distance to the ideotype:


$${\text{MGIDI}}_{\text{i}}=\surd \left(\sum_{\text{j}=1^{\text{f}}}{\left({\text{FS}}_{\text{ij}}-{\text{FS}}_{\text{j}}\text{\_ideal}\right)}^{2}\right)$$

Where FSij is the standardized factor score of genotype i on factor j, FSj_ideal is the corresponding ideotype score, and f is the number of retained factors. The two retained factors explained 54.3% and 28.6% of the total variance, respectively, with a cumulative variance of 82.9%. Smaller MGIDI values indicate closer proximity to the ideotype. Radar plots were used to visualize clone strengths and weaknesses. All analyses were performed using the metan package in R version 4.6.0 (R Core Team, 2026).

### 2.6 Heterotic grouping and breeding recommendations

Heterotic groups were defined using the WAASB stability index and the MGIDI multi-trait performance index. Clones were classified into four groups based on their performance (BLUP estimates for Brix and tillering rate) and stability (WAASB values). Performance thresholds were set at the 25th and 75th percentiles to define high, moderate, and low classes; stability was classified as high or low using the median WAASB as the cutoff. The four resulting groups were: Group A (high performance, high stability), Group B (high performance, low stability), Group C (low performance, high stability), and Group D (moderate performance and stability). All classifications were performed in R version 4.6.0 (R Core Team, 2026) using the metan package.

## 3 Results

The integrated framework (BLUP + AMMI + WAASB + MGIDI) reduced the 100 clones to three elite parents (C059, C086, and C089) that formed Group A (elite + stable), and assigned the remaining clones to three heterotic groups (B, C, D) for use in crossing programs. This classification, supported by genetic parameters, stability indices, and multi-trait selection, is detailed in the following sections.

### 3.1 Breeding values (BVs)

The BLUP-based breeding values (genotypic values) of the 100 clones were examined to characterize genetic diversity, estimate variance components, and identify promising parents. The following subsections present the top-performing clones, heritability estimates, genetic parameters, trait correlations, repeatability, ratooning ability, and relative performance that underpin selection decisions.

#### 3.1.1 Genetic diversity revealed by genotypic values

BLUP-based genotypic values for the 100 clones revealed a wide range across all traits, confirming substantial genetic diversity (**Table 5** (. For juice Brix, Thailand1 (16.11 %), Yunnan2 (16.07 %), and Thailand20032 (14.63 %) ranked top; notably, Thailand1 also ranked among the top ten for stalk weight (113.84g) and exhibited above-average tillering. Likewise, Hainan9250 (124.33g) ranked first for stalk weight, followed by Thailand1 and Hainan9233 (112.18g), while Hainan Yanglan9 (5.02kg) led fresh biomass, ahead of Hainan9225 (3.14kg) and India2 (2.94kg). Meanwhile, the highest tillering rate was observed in Yunnan200934 (1417.42 %), with Hainan Haikou (1250.93 %) and Yunnan82140 (1242.62 %) following closely. In terms of stalk traits, Thailand98T266 (181.15cm) was the tallest, whereas Laos2 (0.83cm) and Yunnan82114 (0.82cm) showed the largest diameter. For internode length, Yunnan76318 (17.47cm) outperformed others, ahead of Yunnan200918 (15.50cm) and Yunnan8277 (15.23cm). Notably, the top-performing clones were predominantly from Yunnan and Hainan, suggesting that these regions harbor particularly valuable genetic diversity for sugarcane breeding. Altogether, these BLUP rankings confirm the wide genetic base and provide a rational, unbiased basis for selecting elite parents for sugarcane pre-breeding programs.

**Table 5:** Top ten *Saccharum spontaneum* clones for seven agronomic traits based on absolute breeding values (BLUP = μ + g_j_), where μ is the overall mean and g_j_ is the random effect of the jth clone.

Rank	Stalk height (cm)		Stalk diameter (cm)		Internode length (cm)		Stalk weight (g)		Fresh biomass (kg)		Juice Brix %		Tillering rate %	
	Clone	BLUP	Clone	BLUP	Clone	BLUP	Clone	BLUP	Clone	BLUP	Clone	BLUP	Clone	BLUP
1	C061	181.15	C052	0.83	C072	17.47	C033	124.33	C062	5.02	C058	16.11	C066	1417.42
2	C075	167.52	C073	0.82	C065	15.50	C058	113.84	C058	3.14	C097	16.07	C044	1250.93
3	C052	167.27	C058	0.79	C084	15.23	C031	112.18	C078	2.94	C060	14.63	C078	1242.62
4	C078	167.24	C100	0.79	C082	14.99	C005	107.18	C005	2.71	C031	14.63	C090	1203.83
5	C091	166.27	C072	0.79	C003	14.70	C073	105.45	C094	2.62	C063	14.55	C100	1167.00
6	C073	165.91	C081	0.78	C083	14.29	C032	103.85	C072	2.61	C051	14.49	C039	1166.75
7	C089	165.19	C064	0.76	C077	14.24	C062	98.03	C008	2.55	C071	14.35	C086	1151.97
8	C094	164.82	C062	0.76	C036	14.01	C018	97.61	C090	2.51	C055	14.27	C047	1088.58
9	C030	164.51	C031	0.76	C069	13.87	C100	96.83	C062	2.48	C046	13.65	C035	1047.24
10	C038	164.02	C066	0.76	C079	13.86	C038	96.55	C058	2.36	C052	13.63	C099	1043.98

Full ranking in **Supplementary Table S1**.

#### 3.1.2 Genetic parameters

Genetic parameters for the seven agronomic traits are summarized in **Table 6**. Genetic variance (σ^2^G) ranged widely, from 0.008 (stalk diameter) and 0.328 (fresh biomass) to 310.05 (stalk height) and 45,280.04 (tillering rate), whereas residual variance (σ^2^ϵ) consistently exceeded σ^2^G for most traits, underscoring the strong environmental influence on trait expression. Broad-sense heritability (H^2^) peaked in stalk diameter (0.40) and stalk height (0.39), both showing moderate expected genetic advance (GAM = 18.10% and 15.87%, respectively), which suggests they are amenable to direct phenotypic selection. In stark contrast, stalk weight displayed the lowest H^2^ (0.12) alongside a substantial GCV (19.98%) and GAM (13.98%), implying that direct selection may be inefficient due to heavy environmental noise. Meanwhile, fresh biomass exhibited low-to-moderate H^2^ (0.25) but the highest GCV (31.62%) and a promising GAM (32.56%), indicating exploitable genetic variability through replicated evaluations; likewise, tillering rate showed moderate H^2^ (0.34) with high GCV (28.15%) and GAM (33.76%), pointing to good potential for genetic gain. Conversely, juice Brix had low H^2^ (0.25) combined with moderate GCV (14.31%) and GAM (14.71%), suggesting that improving sugar content may require marker-assisted selection or multi-environment testing. Notably, GCV% was highest for fresh biomass (31.62%) and tillering rate (28.15%), whereas it was lowest for stalk height (12.40%) and internode length (13.25%). Overall, stalk diameter, stalk height, tillering rate, and fresh biomass are promising targets for breeding, whereas stalk weight and juice Brix will require more careful experimental designs to account for environmental noise.

**Table 6:** Estimates of genetic parameters for seven agronomic traits in 100 *Saccharum spontaneum* clones.

Trait (abbreviation)	σ^2^G	σ^2^E	σ^2^P	μ	H^2^	GCV%	PCV%	GA	GAM%
Stalk height (SH)	310.05	493.44	803.49	141.99	0.39	12.40	19.96	22.53	15.87
Stalk diameter (SD)	0.01	0.01	0.02	0.64	0.40	13.86	21.86	0.12	18.10
Internode length (IL)	2.54	6.89	9.43	12.02	0.27	13.25	25.56	1.70	14.16
Stalk weight (SW)	252.49	1935.73	2188.22	79.53	0.12	19.98	58.82	11.12	13.98
Fresh biomass (FB)	0.33	0.99	1.31	1.81	0.25	31.62	63.25	0.59	32.56
Juice Brix (BX)	2.84	8.54	11.38	11.77	0.25	14.31	28.66	1.73	14.71
Tillering rate (TR)	45280.04	88286.16	133566.20	756.01	0.34	28.15	48.34	255.23	33.76

σ^2^G (genetic variance), σ^2^E (residual variance), σ^2^P (phenotypic variance), μ (overall mean), H^2^ (heritability), GCV% (genetic CV), PCV% (phenotypic CV), GA (genetic advance), GAM% (GA as % of mean). k = 2.06 (5% selection intensity).

#### 3.1.3 Genetic and phenotypic correlations

Genetic and phenotypic correlations (**Figures 2a, b**) were compared for all trait pairs. Strong positive genetic associations emerged between stalk weight and stalk diameter (r_g = 0.59), stalk height and internode length (r_g = 0.57), and stalk height and stalk weight (r_g = 0.42). Similarly, stalk diameter correlated positively with fresh biomass (r_g = 0.46), indicating that thicker stalks contribute to both individual weight and overall biomass. In contrast, a negative genetic correlation was found between juice Brix and internode length (r_g = –0.34), revealing a genetic trade-off between sugar content and stalk elongation. Furthermore, tillering rate showed a moderate positive genetic correlation with fresh biomass (r_g = 0.18), while weak positive associations were also detected between juice Brix and stalk weight (r_g = 0.22), and between stalk height and fresh biomass (r_g = 0.45). As expected, phenotypic correlations were generally lower in magnitude than their genetic counterparts. For instance, the phenotypic correlation between stalk weight and stalk diameter was negligible (r_p = –0.02), whereas the genetic correlation was strongly positive. Nevertheless, some phenotypic associations remained consistent, such as the positive correlations between stalk height and fresh biomass (r_p = 0.42) and between stalk diameter and fresh biomass (r_p = 0.15). Juice Brix showed a weak negative phenotypic correlation with internode length (r_p = –0.12) and a weak positive correlation with stalk weight (r_p = 0.14). Overall, these results confirm that genetic correlations provide a more reliable basis for indirect selection, especially for traits with low heritability such as juice Brix and stalk weight. The negative correlation between Brix and internode length implies that simultaneous improvement of sugar content and stalk elongation will require reciprocal crossing between complementary groups (e.g., high-Brix short-internode × high-biomass long-internode clones), while the positive correlations between stalk diameter and both stalk weight and fresh biomass make stalk diameter a valuable indirect selection criterion.

**Figure 2:**
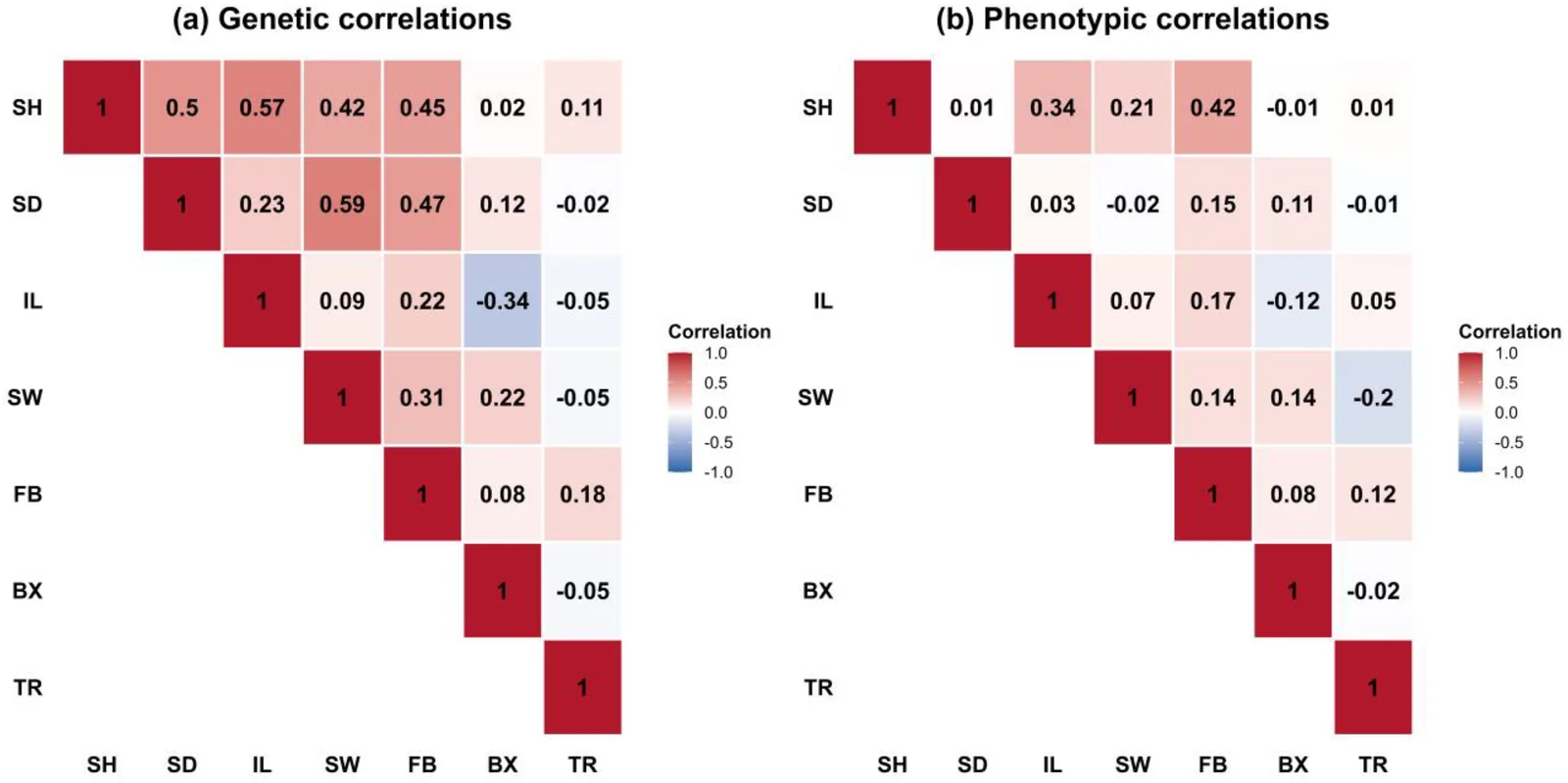
Pairwise genetic [left **(a)**] and phenotypic [right **(b)**] correlations among eight agronomic traits of 100 *Saccharum spontaneum* clones. Only the upper triangle of each correlation matrix is shown. Color intensity and circle size are proportional to the correlation coefficients. Trait stalk height (SH), stalk diameter (SD), internode length (IL), stalk weight (SW), fresh biomass (FB), juice brix (BX), and tillering rate (TR).

#### 3.1.4 Repeatability, ratooning ability, and relative performance

Repeatability estimates between plant cane (PC) and first ratoon (FR), and between FR and second ratoon (SR), were calculated for the seven traits (**Figure 3**). A positive difference (FR-SR minus PC-FR) indicates higher repeatability from FR to SR, whereas a negative difference reflects a decrease in repeatability during the second ratoon. For PC-FR transitions, repeatability ranged from low to high: stalk weight (0.69), tillering rate (0.64), fresh biomass (0.59), stalk diameter (0.56), stalk height (0.46), internode length (0.39), and juice Brix (0.16). The notably low Brix repeatability suggests strong cycle-specific environmental effects, although differences in maturity, harvest timing, or measurement precision may also contribute to this low repeatability. In contrast, FR-SR repeatability was generally very high for most traits: fresh biomass (0.995), juice Brix (0.990), stalk height (0.953), stalk diameter (0.934), and internode length (0.902). Consequently, these traits showed positive differences (FR-SR > PC-FR), with the largest increase for juice Brix (+0.83), followed by stalk height (+0.49) and internode length (+0.51), indicating that clone performance becomes highly stable after the first ratoon.

**Figure 3:**
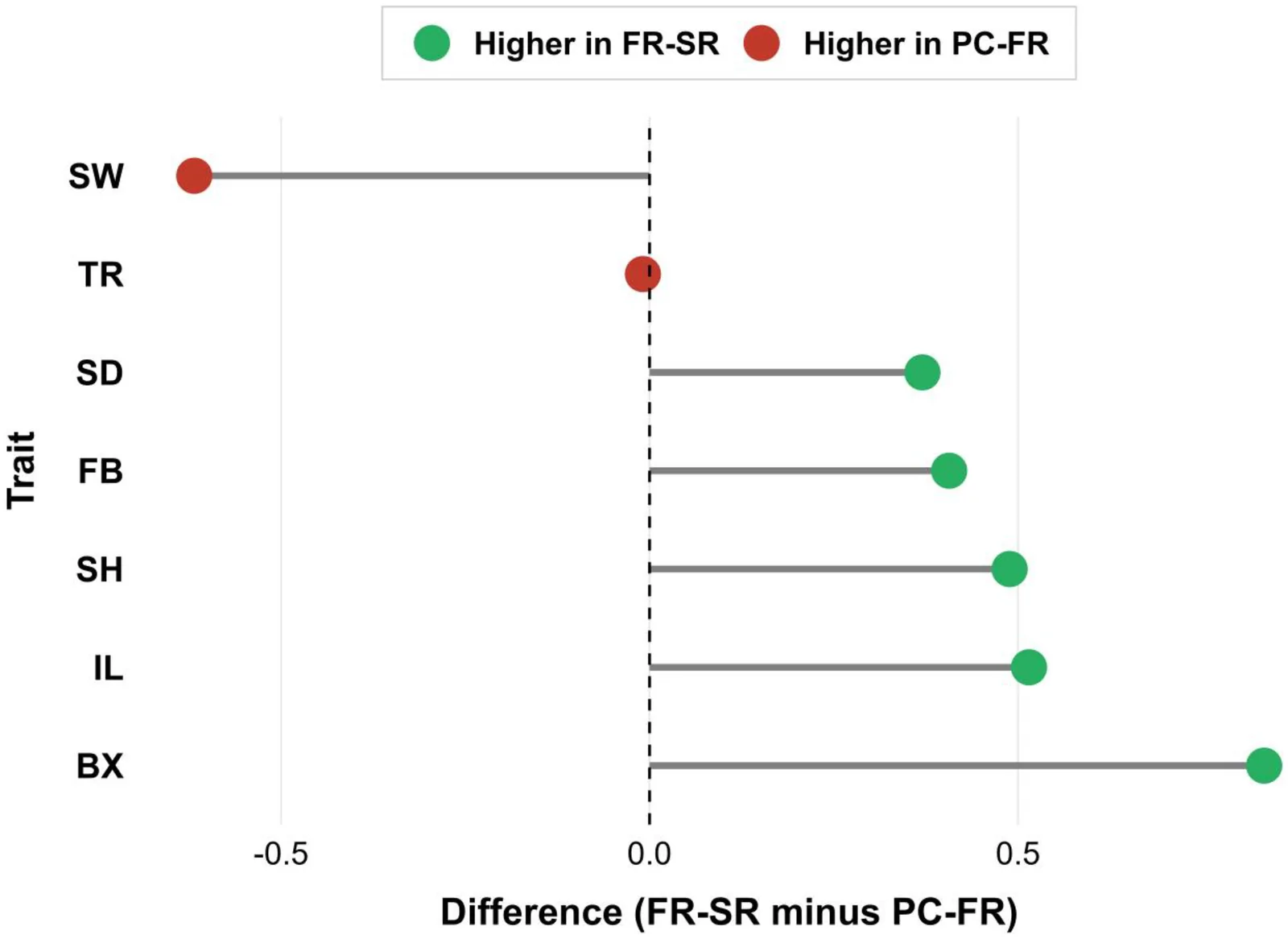
Lollipop chart showing the difference in repeatability (FR-SR minus PC-FR) for seven agronomic traits in *Saccharum spontaneum* clones. Positive differences (green) indicate higher repeatability from first ratoon (FR) to second ratoon (SR), whereas negative differences (red) indicate higher repeatability from plant cane (PC) to first ratoon (FR). The length of each horizontal bar represents the magnitude of the difference, and the dots are colored according to the direction of change. Traits are ordered from top to bottom by decreasing difference. SH, stalk height; SD, stalk diameter; IL, internode length; SW, stalk weight; FB, fresh biomass; BX, juice Brix; TR, tillering rate.

Noteworthy exceptions were stalk weight and tillering rate: stalk weight had a large negative difference (–0.62) due to extremely low FR-SR repeatability (0.07), implying drastic re-ranking in the second ratoon. This may be due to the marked environmental contrasts between crop cycles ( **Supplementary Figure S1**) and soil-related factors, as stalk weight was also the trait with the lowest repeatability; further studies are needed to confirm the underlying causes. Tillering rate showed a small negative difference (–0.01), reflecting a slight decline from moderate PC-FR repeatability (0.64) to moderately low FR-SR repeatability (0.63). Taken together, most yield-related traits (stalk height, diameter, internode length, fresh biomass) achieve excellent FR-SR repeatability, making them reliable selection targets in advanced ratoon generations. Conversely, juice Brix (PC-FR) and stalk weight (FR-SR) require multi-cycle evaluation to avoid genotype-by-ratoon interaction.

The ratooning ability (RA) and relative performance (RP) of the top ten clones were evaluated separately for each of the seven agronomic traits (**Figures 4a, b**, respectively). For RA, most traits displayed values above 100%, indicating that clone performance generally improved or remained stable from plant cane to ratoon cycles. Stalk weight (SW) and fresh biomass (FB) showed the highest RA values, with several clones exceeding 500% (e.g., C053 and C057 in SW; C035 and C055 in FB). In contrast, tillering rate (TR) exhibited RA values close to or slightly below 100% for the top ten clones, suggesting a moderate decline in tillering capacity in ratoon crops. Regarding RP, all traits showed consistent high performance among the selected clones, with mean values ranging from approximately 130% (TR) to over 400% (SW). The highest RP values were recorded for SW (clone C054, 403.3%), FB (clone C037, 231.8%), and stalk height (SH) and internode length (IL) (both >160%). Notably, several clones (e.g., C040, C075, and C020) appeared among the top performers for multiple traits, highlighting their potential as multi-trait donors.

**Figure 4:**
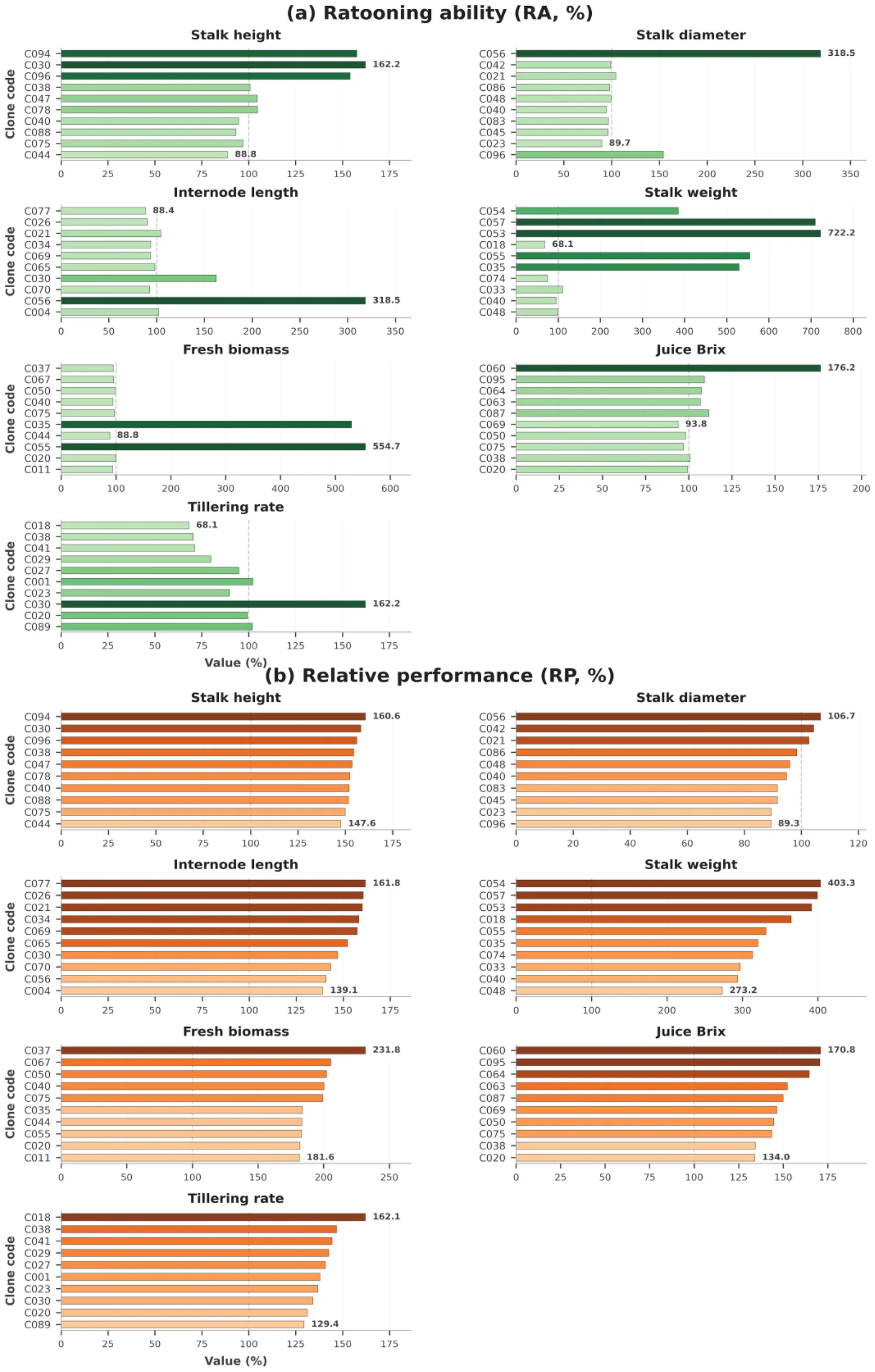
Ranking of the top ten *Saccharum spontaneum* clones for ratooning ability (RA, %) and relative performance (RP, %) across seven agronomic traits evaluated in six environments: **(a)** RA and **(b)** RP. Clone codes within each trait are ordered by rank (highest to lowest). Color intensity indicates the magnitude of the value, with darker green/orange shades representing higher values and lighter shades representing lower values.

### 3.2 Analysis of genotype × environment interaction

While the previous section focused on genetic parameters and performance across ratoon cycles, the following analyses examined stability and performance across the six distinct environments. Climatic characterization across these environments revealed that they clustered primarily by cropping cycle rather than by location, with marked contrasts in precipitation and solar radiation across phenological stages (**Supplementary Figure S1**).

#### 3.2.1 AMMI-based stability and performance

The AMMI-derived indices (ASV, GSI, and HMRPGV) offered complementary insights (**Table 7**): low GSI identified genotypes that successfully combined productivity and stability, whereas high HMRPGV reflected consistent relative performance across environments. For stalk height, C085 (Yunnan8278) exhibited an excellent yield–stability balance (mean = 163.42, ASV = 0.937, GSI = 18), while C043 (Hainan928) was more stable (ASV = 0.594) but with moderate mean (134.94); similarly, C090 (Yunnan84268) combined good mean (152.53) with low ASV (0.772) and GSI (33). Regarding stalk diameter, C072 (Yunnan76318) stood out with high mean (0.80), very good stability (ASV = 0.073), and the lowest GSI (12), whereas C005 (Fujian Putian) and C060 (Thailand20032) also delivered high means (0.73 and 0.76) with comparably low ASV and GSI. In the case of internode length, C091 (Yunnan8727) achieved the highest mean (13.68) while maintaining reasonable stability (ASV = 0.288) and the lowest GSI (25), although C009 (Guangdong28) was the most stable (ASV = 0.028) yet had a lower mean (11.66). For stalk weight, C029 (Hainan9231) and C008 (Guangdong23) showed higher means (0.09 each) with low ASV and GSI (37 each), whereas C099 (Yunnan1) was the most stable (ASV = 0.017) but with low mean (0.08). Concerning fresh biomass, C100 (Yunrui128) achieved the highest mean (2.36) with acceptable stability (ASV = 0.192) and the lowest GSI (18), while C098 (Yunnan10) was more stable (ASV = 0.069) but had a lower mean (1.77). Turning to juice Brix, C058 (Thailand1) proved both the most stable (ASV = 0.068) and highest yielding (16.84), with the lowest GSI (2) and high HMRPGV (0.71); likewise, C055 (Sichuan7929) displayed excellent balance (mean = 14.69, ASV = 0.106, GSI = 10). Finally, for tillering rate, C090 (Yunnan84268) exhibited the highest mean (1252.53) with acceptable stability (ASV = 4.112) and the lowest GSI (12), whereas C091 (Yunnan8727) was the most stable (ASV = 0.340) but with a lower mean (872.94). Overall, low GSI effectively highlighted genotypes that combined productivity and stability across traits: C085 for stalk height, C072 for stalk diameter, C091 for internode length, C008 for stalk weight, C100 for fresh biomass, C058 for juice Brix, and C090 for tillering rate. Meanwhile, high HMRPGV consistently reflected superior relative performance across environments. 

**Table 7:** lists the top ten stable clone per trait, ranked in ascending order of AMMI stability value (ASV), from most stable to least stable, along with their mean performance, first interaction principal component (PC1), genotype selection index (GSI), harmonic mean of relative performance of genotypic values (HMRPGV), and sum of absolute IPC scores (SIPC).

Trait	Rank	Clone code	Mean	PC1	ASV	GSI	HMRPGV	SIPC
Stalk height	1	C043	134.94	0.092	0.594	70.00	1.06	0.597
	2	C029	141.86	-0.184	0.750	59.00	1.00	0.591
	3	C090	152.53	-0.088	0.772	33.00	0.93	0.798
	4	C085	163.42	0.040	0.937	18.00	0.87	0.967
	5	C098	144.72	-0.268	1.096	54.00	0.98	0.868
	6	C041	123.97	-0.248	1.329	87.00	1.15	1.270
	7	C055	140.47	0.297	1.334	66.00	1.02	1.161
	8	C026	144.31	0.400	1.444	58.00	0.99	0.853
	9	C019	131.17	0.426	1.460	82.00	1.09	0.501
	10	C039	122.11	-0.280	1.521	96.00	1.16	1.461
Stalk diameter	1	C097	0.64	0.005	0.023	51.00	1.01	0.021
	2	C032	0.56	0.001	0.026	82.00	1.14	0.027
	3	C005	0.73	0.003	0.040	23.00	0.88	0.042
	4	C078	0.67	0.001	0.044	43.00	0.97	0.045
	5	C055	0.53	0.003	0.052	93.00	1.22	0.054
	6	C006	0.54	0.021	0.070	92.00	1.19	0.032
	7	C072	0.80	0.021	0.073	12.00	0.80	0.048
	8	C026	0.65	0.025	0.082	53.00	0.98	0.040
	9	C060	0.76	-0.015	0.083	24.00	0.84	0.082
	10	C035	0.48	0.001	0.086	108.00	1.34	0.087
Internode length	1	C009	11.66	0.005	0.028	57.00	1.04	0.029
	2	C045	11.17	0.036	0.111	70.00	1.08	0.100
	3	C033	10.65	-0.036	0.153	83.00	1.13	0.159
	4	C043	11.75	-0.082	0.232	56.00	1.03	0.184
	5	C011	9.49	-0.060	0.259	103.00	1.27	0.270
	6	C067	9.61	-0.087	0.287	101.00	1.25	0.268
	7	C091	13.68	0.104	0.288	25.00	0.88	0.217
	8	C007	9.59	-0.021	0.303	104.00	1.25	0.319
	9	C080	11.76	0.026	0.331	60.00	1.03	0.351
	10	C075	11.68	0.111	0.336	64.00	1.03	0.295
Stalk weight	1	C099	0.08	0.005	0.017	47.00	1.17	0.013
	2	C036	0.07	-0.002	0.022	75.00	1.23	0.023
	3	C029	0.09	-0.003	0.023	37.00	0.95	0.023
	4	C013	0.08	-0.002	0.024	61.00	1.13	0.025
	5	C087	0.07	-0.002	0.033	67.00	1.22	0.034
	6	C008	0.09	-0.001	0.033	37.00	0.88	0.034
	7	C003	0.07	-0.012	0.039	79.00	1.28	0.015
	8	C096	0.07	0.006	0.039	72.00	1.14	0.041
	9	C007	0.06	-0.004	0.040	95.00	1.73	0.041
	10	C046	0.07	0.012	0.040	81.00	1.23	0.018
Fresh biomass	1	C098	1.77	0.009	0.069	51.00	1.03	0.038
	2	C013	1.55	-0.011	0.133	71.00	1.20	0.119
	3	C029	1.70	-0.018	0.144	58.00	1.09	0.077
	4	C077	1.95	-0.002	0.150	39.00	0.94	0.151
	5	C088	1.45	-0.018	0.167	80.00	1.28	0.126
	6	C100	2.36	-0.021	0.192	18.00	0.77	0.136
	7	C043	1.36	-0.001	0.195	90.00	1.39	0.196
	8	C027	1.06	0.032	0.268	102.00	1.79	0.165
	9	C018	1.07	0.009	0.295	102.00	1.70	0.297
	10	C011	1.39	0.043	0.326	92.00	1.35	0.145
Juice Brix	1	C058	16.84	0.022	0.068	2.00	0.71	0.080
	2	C055	14.69	0.060	0.106	10.00	0.80	0.094
	3	C072	8.80	0.062	0.106	98.00	1.35	0.088
	4	C012	11.04	0.074	0.168	71.00	1.07	0.188
	5	C084	9.55	0.089	0.213	92.00	1.24	0.242
	6	C049	10.98	-0.020	0.239	76.00	1.07	0.256
	7	C004	12.20	0.147	0.258	50.00	0.97	0.229
	8	C039	13.45	-0.162	0.273	25.00	0.87	0.201
	9	C009	10.64	0.091	0.286	82.00	1.13	0.333
	10	C088	8.40	0.154	0.301	109.00	1.42	0.312
Tillering rate	1	C091	872.94	-0.106	0.340	26.00	0.86	0.123
	2	C050	799.33	-0.230	0.816	40.00	0.94	0.582
	3	C040	410.31	-0.565	1.819	103.00	1.95	0.724
	4	C054	690.86	-0.743	3.025	60.00	1.09	2.608
	5	C005	518.36	-0.820	3.129	96.00	1.50	2.514
	6	C081	880.44	-0.101	3.156	30.00	0.86	3.240
	7	C063	606.97	-0.796	3.393	75.00	1.27	3.031
	8	C090	1252.53	-1.094	4.112	12.00	0.60	3.241
	9	C011	563.14	1.252	4.244	90.00	1.39	2.633
	10	C049	437.25	-1.305	4.541	107.00	1.77	3.067

#### 3.2.2 Parametric stability indices

##### 3.2.2.1 WAASB index

Complementing the AMMI-based stability assessment, the WAASB (weighted average of absolute scores) index was employed to further rank clones for stability across the six environments. Derived from the first three interaction principal components, WAASB integrates the magnitude of IPCA scores weighted by their explained variance; lower values denote higher stability, and the rankings (**Figure 5**) clearly demonstrate that stability is trait-specific. For stalk height, the most stable clone was C043 (Hainan928) with WAASB = 0.130, followed by C019, C090, C029, C085, and C098. Regarding stalk diameter, clone C032 exhibited the lowest WAASB (0.0139), with C055, C006, C067, C005, and C058 completing the top six. Similarly, for internode length, clone C009 (Guangdong28) was the most stable (0.0124), ahead of C045, C033, C067, and C091. Concerning stalk weight, clone C003 ranked first (5.311), followed by C008, C036, C029, and C013. With respect to fresh biomass, clone C098 showed the greatest stability (7.168), ahead of C013, C088, C029, and C011. For juice Brix, the most stable clone was C055 (0.0706), followed by C072, C012, C084, C088, and C049. Finally, for tillering rate, clone C091 was the most stable (0.1670), with C050, C040, C081, C005, and C054 in the top six. Notably, internode length and juice Brix exhibited very low WAASB values (<0.12), reflecting strong genetic control and high stability across environments. In contrast, fresh biomass and stalk weight showed much higher WAASB values (7.17 and 5.31, respectively), indicating their greater environmental sensitivity and the need for multi-environment testing. These trait-specific stability patterns are consistent with the ASV results reported in the previous section, reinforcing the reliability of the WAASB index for ranking clones in this sugarcane germplasm.

**Figure 5:**
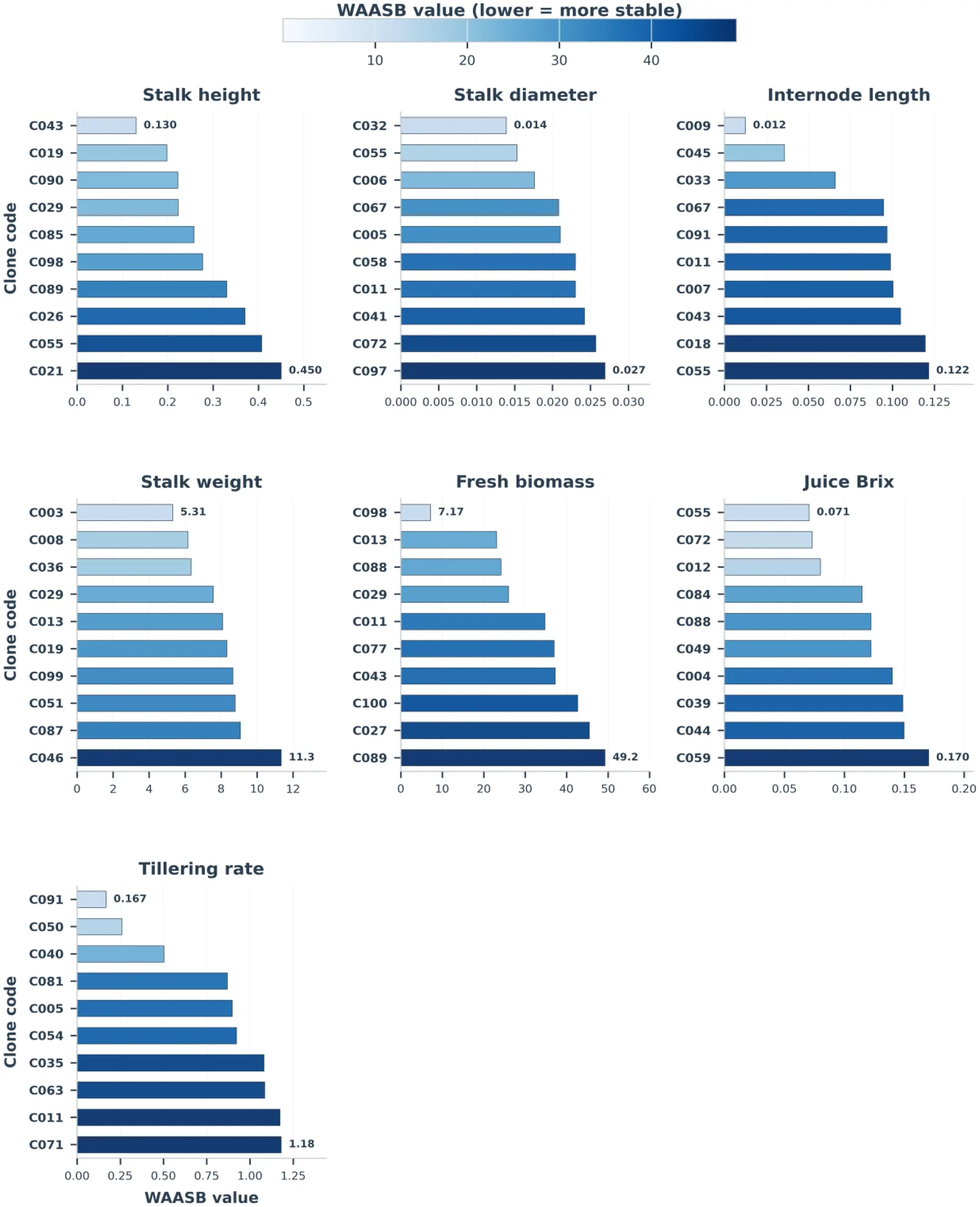
WAASB values of the top ten *Saccharum spontaneum* clones across seven agronomic traits evaluated in six environments. Clones within each trait are ranked from lowest (top) to highest (bottom) WAASB value. Color intensity indicates the magnitude of WAASB, with lighter blue representing lower values (greater stability) and darker blue representing higher values (lower stability).

##### 3.2.2.2 Selection of superior clones based on MGIDI

The MGIDI was applied to simultaneously rank clones across the seven agronomic traits, with the ideotype defined as the maximum value for each trait. The ten clones closest to this ideotype were selected as the best multi-trait performers (**Figure 6**). In the radar plot, clone C018 recorded the lowest MGIDI (9.70), followed by C033 (10.22), C041 (10.90), C049 (10.96), C009 (10.98), C032 (11.02), C008 (11.02), C074 (11.06), C040 (11.12), and C005 (11.19). The black ideotype line (value = 1 for all traits) serves as the benchmark; clones with profiles approaching this line exhibit superior multi-trait balance. For instance, C018 showed consistently high values across most traits, whereas C005, despite ranking within the top ten, exhibited notable weaknesses in stalk height and tillering rate, confirming MGIDI’s ability to distinguish balanced clones from those excelling in few traits.

**Figure 6:**
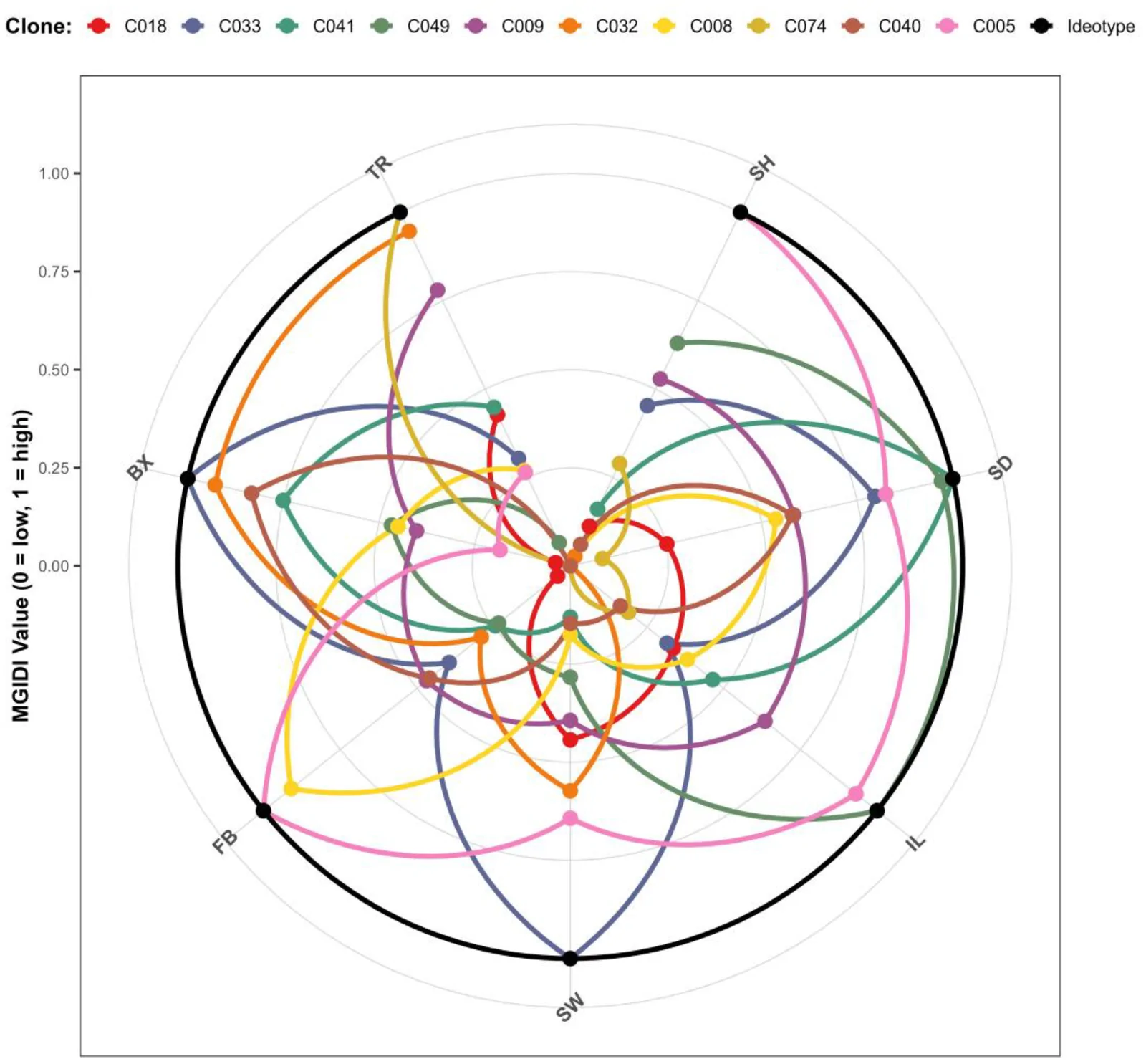
Radar plot of the top ten *Saccharum spontaneum* clones based on MGIDI. Normalized values (0=low, 1=high) are shown for seven traits: SH, stalk height; SD, stalk diameter; IL, internode length; SW, stalk weight; FB, fresh biomass; BX, juice Brix; TR, tillering rate. Each colored line represents one clone (see legend); the black line is the ideotype (maximum value for all traits). Lower MGIDI (closer to center) indicates better overall performance.

### 3.3 Parental heterotic grouping based on MGIDI and WAASB

The MGIDI and WAASB indices were jointly used to classify the 100 clones into four heterotic groups, applying the lower 25th percentile of each index as the threshold for high performance (low MGIDI) and high stability (low WAASB) (**Figure 7**). Remarkably, only three clones, C059, C086, and C089, met both criteria, thereby forming GroupA (elite+stable). For instance, C059 (Thailand20031) ranked 37th in MGIDI and 24th in WAASB, while C086 and C089 also exhibited consistently low ranks for both indices. Consequently, these three clones stand out as the most promising parents, since they simultaneously deliver high yield and quality-related traits alongside reliable ratoon performance. By contrast, GroupB (high performance, low stability) comprised 22 clones, including high-sugar donors such as C058 (Thailand1) and C050 (Hainan Yanglan9), alongside C052, C064, C072, and C100. Although these clones displayed outstanding mean values, their relatively high WAASB values indicate strong environment-dependent performance; therefore, they are best suited for specific favorable environments or should be crossed with GroupA to buffer environmental sensitivity. Similarly, GroupC (low performance, high stability) also contained 22 clones (e.g., C003, C013, C034, C042, C045) that maintained stable rankings across environments yet never reached top performance levels; hence, they are not recommended as parents unless no better alternatives exist. Finally, the remaining 53 clones were assigned to GroupD (moderate), displaying intermediate performance and stability, thus constituting the bulk of the collection and serving to maintain genetic diversity in broad-based crossing blocks. In essence, this MGIDI–WAASB-based classification successfully distilled the initial 100 clones down to three elite multi-trait donors (C059, C086, C089) that combine high performance with broad stability. Accordingly, these clones are now ready for pre-breeding programs aimed at developing climate-resilient, high-sugar, and high-biomass sugarcane hybrids (full ranking in **TableS1**).

**Figure 7:**
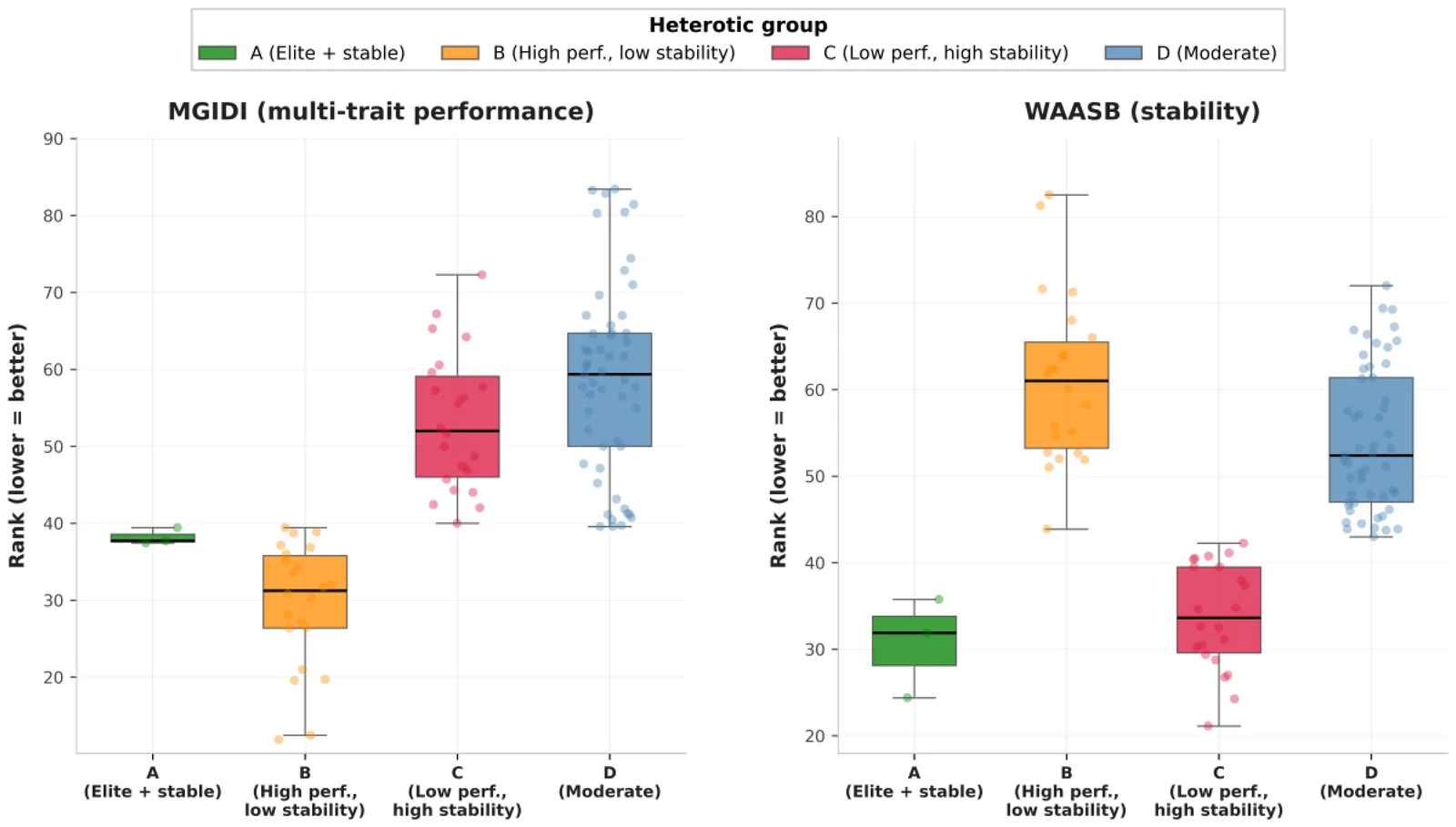
Heterotic grouping of 100 *Saccharum spontaneum* clones based on multi-trait performance (MGIDI) and stability (WAASB) across seven agronomic traits evaluated in six environments. Boxplots show the distribution of ranks for each heterotic group: A (green; elite + stable), B (orange; high performance, low stability), C (red; low performance, high stability), and D (blue; moderate). Individual data points are overlaid as jittered dots. Lower rank indicates better performance/stability.

## 4 Discussion

### 4.1 Genetic architecture

Genotypic values (μ+g_j_) showed a wide range, confirming substantial diversity within this S. spontaneum collection (Piepho et al., 2008; Vigneshwari et al., 2025). The wide genetic diversity observed in this Asian S. spontaneum collection aligns with the findings of Xu et al. (2020) for a Chinese core collection. Unlike previous work, this study incorporates stability ty and multi-trait selection indices, thereby offering a more integrated pre-breeding framework for parental selection and heterotic grouping. Although BLUP estimates of the best-performing clones clustered closely, a consequence of selecting only the uppermost fraction of the distribution, this narrow range reflects selection effectiveness rather than a lack of genetic variation (Abu-Ellail et al., 2026). Moderate heritability for stalk diameter (H^2^ = 0.40) and stalk height (H^2^ = 0.39) indicates reasonably strong genetic control, making them responsive to direct phenotypic selection (Abu-Ellail et al., 2019; Pathy et al., 2025; Saadan et al., 2025); accordingly, their moderate expected genetic advance (GAM = 18.10% and 15.87%) supports early-generation screening. Conversely, juice Brix and stalk weight exhibited low heritability (0.25 and 0.12), reflecting strong environmental influence typical of wild S. spontaneum (Ngaklunchon et al., 2024), and thus requiring multi-environment testing or indirect selection via correlated traits.

Ratooning ability (RA) was high for stalk weight and fresh biomass, confirming that these clones maintain productivity across cycles (Abu-Ellail et al., 2019);clone C040 appeared among the top ten for five traits, supporting ratoon-oriented selection (Saadan et al., 2025). The capacity to sustain productivity under repeated ratooning and environmental stress is a complex trait, involving both physiological resilience and genetic tolerance to abiotic stresses such as drought and temperature extremes (Srivastava and Kumar, 2020). PC-FR repeatability was low for Brix (0.16) and moderate for most yield traits, whereas FR-SR repeatability was remarkably high (≥0.90) for stalk height, diameter, internode length, and fresh biomass (Abu-Ellail et al., 2019), aligning with the FR-SR Brix (0.99) reported by Masri and Amein (2015). Hence, selection for most biomass traits can be based on first-ratoon performance, shortening the breeding cycle. Nevertheless, stalk weight showed extremely low FR-SR repeatability (0.07), indicating drastic re-ranking in the second ratoon (Abu-Ellail et al., 2018), while tillering rate displayed moderate repeatability (0.63), agreeing with Gomathi et al. (2013) that the decline varies among genotypes; both traits thus require multi-cycle evaluation. The high FR-SR repeatability for morphological traits aligns with Jackson (1994), confirming that selection in advanced ratoons is reliable. In sharp contrast, juice Brix had the lowest PC-FR repeatability (0.16), much lower than values reported by Singh and Singh (1999), likely due to cycle-specific environmental effects—hence, multi-cycle evaluation is also essential for Brix.

Regarding trait correlations, the strong positive genetic correlation between stalk weight and stalk diameter (r_g = 0.59) confirms that stalk diameter is an effective indirect selection criterion for stalk weight (Xu et al., 2017; Saadan et al., 2025). Moreover, the negative correlation between juice Brix and internode length (r_g = –0.34) reflects a well-known trade-off (Pan et al., 2004; Jackson, 2005), implying that simultaneous improvement requires reciprocal crossing between complementary groups (e.g., high-Brix short-internode × high-biomass long-internode clones). Tillering rate showed a positive correlation with fresh biomass (r_g = 0.18) but a large residual variance, indicating strong micro-environmental effects; therefore, multi-environment evaluation is essential before using tillering as a selection criterion. Overall, stalk diameter and stalk height are the most promising for direct selection, whereas juice Brix, stalk weight, and tillering rate require multi-environment testing, stability analysis, and multi-trait indices for effective parental selection (Masri et al., 2015; Repale et al., 2024).

### 4.2 Stability patterns

The large environmental variance observed is typical for sugarcane, where yield and quality are strongly modulated by rainfall, temperature, and soil conditions (Meena et al., 2024). In contrast, the smaller genotypic variance indicates a narrow genetic base; however, significant GEI reveals that even elite clones re-rank across environments, reinforcing the need for thorough stability assessment (Durai et al., 2025). ASV patterns were trait-specific: stalk diameter and weight showed low ASV (stronger genetic control), whereas juice Brix and tillering rate showed much higher ASV (greater sensitivity to water and temperature), consistent with previous reports (Appunu et al., 2024; Durai et al., 2025). Moreover, the trade-off between high mean performance and low stability is common; accordingly, GSI penalized unstable high-yielding clones while rewarding those with acceptable yield across environments (Durai et al., 2025; Liao et al., 2026). Clones with high HMRPGV but modest yield performed near the environmental mean (valuable for marginal areas), whereas low-HMRPGV clones were more responsive to improved conditions. Thus, combining ASV and HMRPGV gives richer adaptation insights, while the strong GEI for Brix and tillering rate, consistent with the drier, warmer second-ratoon conditions, necessitates testing across a wide water/temperature range (Liao et al., 2026). Furthermore, older ratoons experienced warmer and drier conditions during grand growth and ripening, which can negatively affect sucrose accumulation (Milligan et al., 1996; Bonnett et al., 2006).

### 4.3 Breeding pipeline

MGIDI computes Euclidean distance to a predefined ideotype (maximum trait values), respecting correlations without arbitrary weights (Olivoto and Nardino, 2021). Hence, the ten lowest-MGIDI clones approached the ideal profile: C018 performed best across traits, whereas C005 (highest within the top ten) showed weaker overall performance, confirming MGIDI’s ability to distinguish balanced clones from those excelling in few traits (Appunu et al., 2024; Adilakshmi et al., 2025; Vinu et al., 2025). Similarly, WAASB (Olivoto et al., 2019) was equally effective for ranking clones, though stability was notably trait-dependent. For instance, C043 combined good yield with broad adaptation, while C032 (stalk diameter) had moderate yield yet high stability; notably, C009 showed exceptionally low WAASB for internode length, possibly due to an efficient root system (Martiné et al., 2001). In contrast, stalk weight exhibited high WAASB, but C003 achieved moderate stability. Furthermore, C098 led in fresh-biomass stability, C055 was stable and high-yielding for Brix, and C091 was most stable for tillering rate; clones ranking top-ten for multiple traits (e.g., C055, C011) are particularly valuable as parents. Joint MGIDI-WAASB classification grouped the 100 clones into four heterotic groups, with only three clones passing both thresholds to form GroupA (elite+stable), consistent with Adilakshmi et al. (2025), who selected only two near-ideals from 11. Conversely, GroupB (high performance, low stability) included 22 clones (e.g., C058, C050) that thrive only under favorable conditions; however, crossing them with GroupA can buffer environmental sensitivity (Milligan et al., 1996). This mirrors Appunu et al. (2024), who also identified only three superior genotypes out of 21. Regarding the rest, GroupC (low performance, high stability) contains 22 clones not recommended as parents, whereas GroupD (moderate) comprises 53 clones useful for maintaining diversity. Following Cooper et al. (2014), crossing should prioritize GroupA × GroupA to fix elite traits and GroupA × GroupB to combine stability with specific adaptation. Ultimately, this integrated framework transformed the initial 100 clones into a structured pre-breeding pipeline. The results offer a clear roadmap for breeders: (i) deploy GroupA clones (C059, C086, C089) as immediate parents for broad adaptation; (ii) cross GroupA × GroupB to combine stability with high yield; (iii) utilize GroupD to safeguard genetic diversity; and (iv) exclude GroupC unless absolutely necessary. Collectively, these data-driven recommendations provide a robust strategy for harnessing wild S. spontaneum germplasm to develop climate-resilient, high-sugar, and high-biomass sugarcane hybrids.

## 5 Conclusion

This study successfully demonstrated the effectiveness of an integrated multi-trait stability framework combining BLUP breeding values, AMMI-based stability indices (ASV, GSI, HMRPGV, WAASB), and the MGIDI selection index for screening large collections of wild *S. spontaneum* germplasm. The framework reduced 100 clones to three elite stable clones (C059, C086, and C089) forming Group A (elite + stable), and classified the remaining clones into three heterotic groups (B, C, D) to guide systematic crossing strategies. Stability was trait-specific, with stalk diameter and stalk height showing low ASV and high repeatability from first to second ratoon (≥0.90), whereas stalk weight and juice Brix exhibited low repeatability and required multi-cycle evaluation. The WAASB-MGIDI classification provided a clear heterotic structure, with Group A clones recommended as immediate parents and Group A × Group B crosses recommended to combine stability with high performance. Geographically distant parents (e.g., Yunnan × Hainan) should be prioritized to maximize heterosis. While these results are robust for pre-breeding, further validation across wider environments is needed. The integrated framework presented here offers a practical, ready-to-use strategy for sugarcane breeders, facilitating the systematic utilization of wild *S. spontaneum* germplasm in climate-adaptive breeding programs.

## Data Availability

The original contributions presented in the study are included in the article/**Supplementary Material**. Further inquiries can be directed to the corresponding authors.
