# Supplementary material for: Breeding values, stability, and heterotic grouping of Asian Saccharum spontaneum collection for parental selection in sugarcane program
Source: Front Plant Sci. 2026 Sep 28;17:1923174. doi: 10.3389/fpls.2026.1923174 (PMC13656251; doi:10.3389/fpls.2026.1923174)
Supplement: Supplementary file 1 [file Table2.doc]

**Supplementary Figure S1.** Heatmap of six standardized climate variables (precipitation, temperature, GDD base 10 °C, humidity, solar radiation, and wind speed) across six environments (two locations × three crop cycles) and four phenological stages (I–IV). Values are scaled per variable (blue = below mean, red = above mean). Dendrograms show clustering of variables (top) and environments (left). Environments clustered by cropping cycle: plant cane (high precipitation/radiation in stages III–IV), first ratoon (intermediate), and second ratoon (highest GDD/temperature, lowest precipitation). Stages I–II were cooler/drier, while stages III–IV showed higher temperature, GDD, and radiation.
